# COVID-19 Rapid Antigen Tests: Bibliometric Analysis of the Scientific Literature

**DOI:** 10.3390/ijerph191912493

**Published:** 2022-09-30

**Authors:** Andy Wai Kan Yeung, Emil D. Parvanov, Faisal A. Nawaz, Rehab A. Rayan, Maria Kletecka-Pulker, Harald Willschke, Atanas G. Atanasov

**Affiliations:** 1 Oral and Maxillofacial Radiology, Applied Oral Sciences and Community Dental Care, Faculty of Dentistry, The University of Hong Kong, Hong Kong, China; 2 Ludwig Boltzmann Institute Digital Health and Patient Safety, Medical University of Vienna, 1090 Vienna, Austria; 3 Department of Translational Stem Cell Biology, Research Institute of the Medical University of Varna, 9002 Varna, Bulgaria; 4 College of Medicine, Mohammed Bin Rashid University of Medicine and Health Sciences, Dubai P.O. Box 505055, United Arab Emirates; 5 Department of Epidemiology, High Institute of Public Health, Alexandria University, Alexandria 5424041, Egypt; 6 Institute for Ethics and Law in Medicine, University of Vienna, Spitalgasse 2-4, 1090 Vienna, Austria; 7 Department of Anaesthesia, Intensive Care Medicine and Pain Medicine, Medical University Vienna, Waehringer Guertel 18-20, 1090 Vienna, Austria; 8 Institute of Genetics and Animal Biotechnology of the Polish Academy of Sciences, 05-552 Jastrzebiec, Poland

**Keywords:** COVID-19, SARS-CoV-2, coronavirus, rapid antigen test, lateral flow test, public health surveillance, saliva, nasopharyngeal swab, nasal swab, pandemic

## Abstract

As the COVID-19 pandemic continues to disrupt health systems worldwide, conducting Rapid Antigen Testing (RAT) at specified intervals has become an essential part of many people’s lives around the world. We identified and analyzed the academic literature on COVID-19 RAT. The Web of Science electronic database was queried on 6 July 2022 to find relevant publications. Publication and citation data were retrieved directly from the database. VOSviewer, a bibliometric software, was then used to relate these data to the semantic content from the titles, abstracts, and keywords. The analysis was based on data from 1000 publications. The most productive authors were from Japan and the United States, led by Dr. Koji Nakamura from Japan (*n* = 10, 1.0%). The most academically productive countries were in the North America, Europe and Asia, led by the United States of America (*n* = 266, 26.6%). Sensitivity (*n* = 32, 3.2%) and specificity (*n* = 23, 2.3%) were among the most frequently recurring author keywords. Regarding sampling methods, “saliva” (*n* = 54, 5.4%) was mentioned more frequently than “nasal swab” (*n* = 32, 3.2%) and “nasopharyngeal swab” (*n* = 22, 2.2%). Recurring scenarios that required RAT were identified: emergency department, healthcare worker, mass screening, airport, traveler, and workplace. Our bibliometric analysis revealed that COVID-19 RAT has been utilized in a range of studies. RAT results were cross-checked with RT-PCR tests for sensitivity and specificity. These results are consistent with comparable exchanges of methods, results or discussions among laboratorians, authors, institutions and publishers in the involved countries of the world.

## 1. Introduction

The COVID-19 Rapid Antigen Test (RAT) is now a frequently investigated theme in the research community, given that the COVID-19 pandemic has been affecting many countries around the world since 2020 [1]. To date, some countries have fully re-opened or are planning to reopen their borders to travelers. However, transmission across country borders increases when travel of contagious persons across country borders increases. Even for a highly vaccinated population, the comorbidities of patients with COVID-19, the impact of long COVID-19, and long-term post-COVID-19 disabilities collectively amount to a substantial toll on the healthcare systems [2]. Abrupt episodes of lockdown may be opted for by some authorities as part of life-saving efforts to contain the pandemic [3]. Another measure is to perform RAT at a border to identify contagious persons, who can be subsequently quarantined [4]. A test showing no antigen could decrease the time in isolation of a contagious person. For these reasons, studying the use of rapid antigen testing by countries world-wide could yield useful information.

COVID-19 vaccination is an effective measure to lower the disease severity and transmission rate [5,6,7]. Besides vaccination, performing diagnostic tests for COVID-19 at regular intervals can identify cases and pre-emptively stop the transmission chain. Though still debatable, the real-time Reverse Transcription Polymerase Chain Reaction (RT-PCR) has been used as a gold standard in many institutions and research studies [8]. However, such tests need to collect the upper respiratory specimen sampled from the subject and transfer it to the laboratory for processing, yielding results after several hours. Therefore, the RAT has been developed for large-scale utilization, so that subjects can perform the self-test on a regular basis with a quick result and without the need to consume laboratory capacity. It has been estimated that through mass testing in workplaces in Spain, the economic impact was EUR 10.44 per test performed, or EUR 5575.49 per positive case detected, with the savings mainly derived from better use of health resources and improved health rates due to subsequent prevention of patient morbidity and mortality [9]. A study in Barcelona estimated that the benefit–cost ratio of doing a RAT was 1.63 [10]. Meanwhile, the timing and frequency of performing the RAT should be considered carefully. The RAT has a lower analytic sensitivity compared to RT-PCR, so its results can only be positive when the viral load is significantly high, presumably reaching the infectious stage of the virus trajectory [11]. Hence, there are debates on the roles of RAT and RT-PCR, such as in the routine airline testing [12].

The broad and diverse body of scientific literature on the COVID-19 RAT may create difficulties for researchers in identifying the major themes, the entities with the heaviest contributions, or the journals in which to look for relevant publications on the subject. Bibliometrics provide an analytical method to synthesize a quantitative overview of the entire research literature on specific topics [13]. This bibliometric analysis of COVID-19 RAT literature aimed to identify and elaborate on the major topics and scientometric characteristics within this body of literature, and enable insights for future research directions. The objectives were to identify the most productive authors, institutions, countries/regions, journals, as well as the most frequently recurring terms and keywords. To the best of our knowledge, no such analysis has been published to date.

## 2. Materials and Methods

On 6 July 2022, the Web of Science (WoS) Core Collection electronic literature database was queried with the following search string: TS = (COVID* OR “SARS-COV-2*”) AND TS = (“antigen test*” OR “self test*” OR “lateral flow test*”). The “TS” operational code involves searching within the title, abstract, and keywords. The asterisk (*) means truncation, and it allows the search to include word variants that begin with the search words. Publications tagged as “Early Access” (*n* = 59) were excluded, as they contained incomplete pagination information and could not be processed by VOSviewer. Additional filters were not applied to limit the search results (e.g., no limitation was applied to the publication language). The search resulted in 1000 publications to be analyzed. The Analyze Results and Citation Report functions of the WoS platform were used for basic frequency count and calculation of the citations per publication (CPP) of the most productive authors, institutions, countries, journals, and WoS journal categories.

The complete record and cited references of these 1000 publications were subsequently loaded into VOSviewer as tab delimited files for generating a term map. A term map relates the publication and citation data to the terms appearing in the titles and abstracts of the publications. Each term is visualized as a circle, with its color indicating the Citations Per Publication (CPP) mentioning that particular term, and its size indicating the publication count. The distance between two circles indicates the frequency of co-occurrence of the two terms within the 1000 publications. For visualization clarity, only terms that appeared in at least 1% of the literature set (that is, a minimum of *n* = 10) were included in the term map. VOSviewer was also used to identify the top 20 recurring author keywords. The study design was similar to prior bibliometric studies conducted by the authors [14,15,16].

## 3. Results

Since the COVID-19 pandemic started, a total of 1000 publications concerning COVID-19 RAT have been published. There were 80 publications in 2020, 650 publications in 2021, and 270 publications in 2022 so far (6 July). Most of the publications (79.7%) were original articles, whereas review papers accounted for another 7.9%. Letters and editorial materials accounted for 5.2% and 3.0%, respectively.

Table 1 lists the top five most productive entities (with the highest publication count) in terms of authors, institutions, countries, journals, and journal categories, respectively. The most productive author was Dr. Koji Nakamura from Tsukuba Medical Center Hospital, Japan. Other authors with at least eight publications were also based in Japan or at the Centers for Disease Control and Prevention (CDC) in the United States. Regarding the most productive institutions, they were based in the United States, the United Kingdom, and Germany. Similarly, the five most productive countries/regions were the United States, England, Germany, India, and Italy. Japan ranked 6th (not listed in Table 1, *n* = 59, CPP = 11.6). There seemed to be no single clearly dominant journal or journal category, but the publications were preferably published in journals concerning infectious diseases, general and internal medicine, public environmental and occupational health, microbiology, and virology.

**Figure 1:**
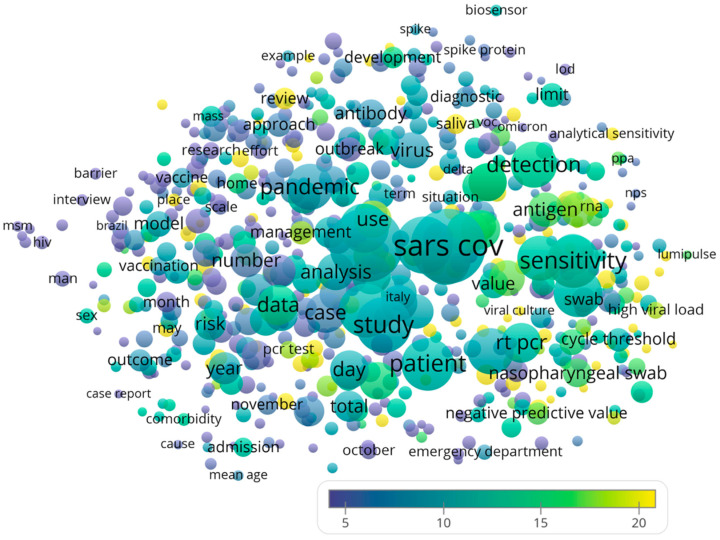
Term map generated using VOSviewer showing recurring terms in the titles and abstracts of the literature concerning the COVID-19 rapid antigen test. Circle size indicates publication count. Circle color indicates citations per publication (CPP). Distance between circles indicates how frequently the two terms co-occurred within the analyzed literature.

From the term map in Figure 1, it may be observed that some of the most frequently recurring terms included sensitivity (*n* = 373, CPP = 12.4) and detection (*n* = 288, CPP = 14.1). When the terms were further examined, the following points could be revealed. Regarding the common sampling methods, “saliva” was mentioned more frequently (*n* = 54, CPP = 21.8) than “nasal swab” (*n* = 32, CPP = 7.9) and “nasopharyngeal swab” (*n* = 22, CPP = 12.3). Regarding the scenarios that required a RAT, several terms could be identified: emergency department (*n* = 31, CPP = 8.5), healthcare worker (*n* = 30, CPP = 7.3)/health care worker (*n* = 13, CPP = 47.9), mass screening (*n* = 20, CPP = 17.4), airport (*n* = 15, CPP = 9.3), workplace (*n* = 15, CPP = 8.4), and traveler (*n* = 11, CPP = 11.5). By inspection of Figure 1, there was no obvious separation or aggregation of bibliometric search terms. This result is consistent with equivalent exchanges of methods, results or discussions among the researchers, authors and publishing companies in the involved countries in the world.

The top 20 author keywords are listed in Table 2. Sensitivity and specificity were on the list, suggesting that the accuracy of the RAT was among the topics of greatest concern. RT-PCR and PCR were also on the list, suggesting that consistent diagnostic accuracy with the PCR tests was also considered important.

## 4. Discussion

The COVID-19 pandemic is a global public health issue. Quick and reliable diagnostic testing is constantly demanded. The WHO recommends that any COVID-19 RAT should meet the minimum performance requirements of ≥80% sensitivity and ≥97% specificity [17]. Consequently, sensitivity and specificity were found to be among the most frequently recurring terms in the titles and abstracts of the analyzed literature, as well as in the author keywords. One of the most highly cited publications identified in this bibliometric analysis was a Cochrane review conducted by Dinnes et al. in 2020 [18] (497 citations), which meta-analyzed eight evaluations of RAT from five studies to compute an average sensitivity of 56.2% and specificity of 99.5%. It is worth mentioning that its updated version in 2021 [19] (eight citations) analyzed 58 evaluations of RAT from 48 studies and reported much more detailed analyses showing that symptomatic participants had higher average sensitivity compared to asymptomatic participants (72.0% vs. 58.1%). Average sensitivity was higher in the first week after symptoms onset than in the second week of symptoms (78.3% vs. 51.0%). Sensitivity was higher in those with cycle threshold (Ct) values on PCR ≤ 25 (higher viral load) compared to those with Ct values > 25 (94.5% vs. 40.7%). Overall average specificity was 99.6%.

Regarding the studies that required RAT, this bibliometric analysis identified a high frequency of mentions of emergency department, healthcare worker, mass screening, airport/traveler, and workplace. In the emergency department with many patients and healthcare workers staying in a relatively confined area, it is essential to promptly identify patients with COVID-19 so that they can be put under isolated care to limit further transmission. Many earlier studies during the severe pandemic periods were conducted with samples from the emergency departments of hospitals. For example, during a 4.5-month period in late 2020, 6.5% of patients attending to the emergency department of an Italian hospital were diagnosed as COVID-19-positive [20]. Since time is the key parameter associated with emergency department functioning, and RAT is easy to use and yields results quickly, it was advocated to test patients with RAT first, with the results to be confirmed later on by the gold standard diagnostic of RT-PCR [20]. Meanwhile, it was found that at one point COVID-19 prevalence reached 32.8% in the emergency department of a German university hospital in early 2021, with the sensitivity of RAT reaching 72.0–75.3% whereas its specificity reached 99.4–100% [21]. It should be noted that some researchers were more cautious about the use of RAT, particularly since a Swiss study on asymptomatic patients admitted to the emergency department found that RAT could only detect COVID-19 in two out of seven patients who later tested positive by RT-PCR [22].

Healthcare workers are more susceptible to COVID-19 infection when they perform invasive or aerosol-generating procedures on patients who are COVID-19-positive. Case reports have been published on mini-outbreaks or cluster infections concerning healthcare workers. One example was the infection with the Alpha variant (B.1.1.7) by two physicians and one nurse working on the same shift, with the two physicians being fully vaccinated with the Pfizer-BioNTech (two shots) one month prior to symptom onset [23]. The infection was detected by RAT and confirmed by RT-PCR and serological tests. Some argued that daily RAT is a viable alternative to mandatory vaccination, particularly targeting health and social care workers in some countries [24]. During this pandemic era, healthcare workers need to stay vigilant to contain the spread of COVID-19, including the use of FFP2 masks instead of surgical masks if they have frequent exposure to COVID-19 patients [25].

Mass screening can be beneficial or essential to safeguard the population from getting COVID-19 on a large scale. One Spanish report suggested that after testing negative by RAT, people that subsequently attended a live music concert did not have an increased risk of COVID-19 infection compared to people that did not attend the event [26]. The study concluded that with adequate protective measures, including RAT screening before entrance, sociocultural activities with a large crowd could also be safe. Testing before the event was also documented and evaluated for other events, such as business conferences, sports events, festivals, and religious events [27].

Mass screening of travelers at the point of entry of a city, such as an airport or the port, may be essential for some regions in terms of public health considerations or to reopen their borders [28,29]. Before samples are collected, it is advised not to eat, drink, gargle, or smoke in advance [28]. It may be difficult to confirm the compliance of so many people at the point of entry, and equally difficult to ensure all positive patients are detected and confined (especially in asymptomatic cases). COVID-19 transmission during travel should also be considered, as multiple cases of in-flight transmission of COVID-19 have been reported in the literature [30]. As a result, some airlines now request RAT negative results from their passengers before boarding flights [30].

Some workplaces request employees to conduct RAT in specific intervals (e.g., daily, or once every 2–3 days) and to enter their workplace only if they had tested negative. The CDC suggests that the frequency of testing could depend on the severity of the pandemic, such as cumulative incidence during the past 7 days and test positivity rate for the local community [31]. Mandatory RAT before coming to work can be a controversial issue that concerns public health and can have major social impacts. Notwithstanding, smartphone apps have been invented to store user-reported COVID-19 RAT data, together with proximity and contact tracing data, to alert employees to maintain social distancing within the workplace [32]. Additionally, it has been argued that the false positive rate of RAT was very low and led to a reduced likelihood of unnecessary workplace disruptions while offering the potential to pre-emptively break transmission chains [33].

Saliva was mentioned more frequently than the nasal swab and nasopharyngeal swab. Many studies have compared the diagnostic accuracy of these samples. For instance, a Japanese study on 10 patients with COVID-19 reported that the nasopharyngeal swab had the highest sensitivity (100%) compared to the nasal swab (67.5%) and saliva (37.5%), but simultaneously, the nasopharyngeal swab had the lowest specificity (52.9%) compared to nasal swab (76.5%) and saliva (94.1%) [34]. Meanwhile, a meta-analysis showed that the sensitivity and specificity of saliva samples was 83.2% and 99.2% respectively, compared to 84.8% and 98.9% for nasopharyngeal swabs respectively, implying that saliva sample could be a good alternative to the nasopharyngeal swab [35]. Another study estimated that using the nasopharyngeal swab could detect 79 more positive cases than saliva in a 100,000 population with the COVID-19 prevalence rate being 1%, but with the incremental cost per additional positive case identified being USD 8093 [36]. Considering this, saliva seemed to be a viable alternative to the nasopharyngeal swab, given that saliva samples could be collected without trained personnel.

This work represents the first total-scale bibliometric analysis of the RAT scientific literature. While the yielded results reveal specific patterns and characteristics associated with the respective research area, readers should be aware of several limitations. WoS does not cover all academic literature, and hence some publications are unavoidably missed. Other databases also have their own drawbacks. For instance, erroneous data in Scopus has been documented in detail [37]. Meanwhile, publication and citation counts may have been seen as surrogates of scientific impact, but they are not equivalent to scientific quality. Despite these limitations, the presented data enable readers to gain an understanding of the recurring themes of the literature on COVID-19 RAT.

## 5. Conclusions

This work identified 1000 publications on COVID-19 RAT published since 2020 (until the time of the query, 6 July 2022). The most academically productive authors were from Japan and the United States. Besides the latter countries, the most productive countries were in Europe and Asia. Publications were preferably published in journals concerning infectious diseases, general and internal medicine, public environmental and occupational health, microbiology, and virology. Sensitivity and specificity were among the most frequently recurring author keywords. Regarding the common sampling methods, saliva was mentioned more frequently than the nasal swab and nasopharyngeal swab, and regarding the scenarios that required RAT, several terms could be identified: emergency department, healthcare worker, mass screening, airport, traveler, and workplace. The published literature indicates broad-scale international public utilization of COVID-19 RAT. Further research is warranted to explore the feasibility of such testing in diverse settings.

## Figures and Tables

**Table 1:** Top five most productive entities in terms of authors, institutions, countries, journals, and journal categories of the literature concerning the COVID-19 rapid antigen test (N—number of publications).

Entities	N (%)	Citations per Publication (CPP)
Authors (6 authors co-ranked 3rd)		
Nakamura, Koji	10 (1.0)	4.0
Kirking, Hannah L	9 (0.9)	12.9
Akashi, Yusaku	8 (0.8)	4.1
Notake, Shigeyuki	8 (0.8)	4.1
Suzuki, Hiromichi	8 (0.8)	4.1
Takeuchi, Yuto	8 (0.8)	4.1
Tate, Jacqueline E	8 (0.8)	14.5
Ueda, Atsuo	8 (0.8)	4.1
Institutions (2 of them co-ranked 5th)		
University of California system	37 (3.7)	7.5
University of London	37 (3.7)	14.4
Centers for Disease Control and Prevention (CDC, USA)	25 (2.5)	11.9
Harvard University	24 (2.4)	44.4
German Center for Infection Research	22 (2.2)	19.3
Imperial College London	22 (2.2)	6.7
Countries/regions		
United States of America	266 (26.6)	10.9
England	120 (12.0)	15.6
Germany	98 (9.8)	8.9
India	70 (7.0)	3.7
Italy	62 (6.2)	10.1
Journals		
Diagnostics	35 (3.5)	3.6
International Journal of Infectious Diseases	32 (3.2)	15.8
Journal of Clinical Virology	27 (2.7)	40.0
BMJ British Medical Journal	24 (2.4)	7.6
PLoS ONE	23 (2.3)	3.8
Journal categories		
Infectious Diseases	210 (21.0)	10.8
Medicine General Internal	204 (20.4)	11.1
Public Environmental Occupational Health	112 (11.2)	3.6
Microbiology	107 (10.7)	13.6
Virology	78 (7.8)	18.0

**Table 2:** Top 20 author keywords in the literature concerning the COVID-19 rapid antigen test (N—number of publications).

Author Keyword	N (%)	Citations per Publication (CPP)
COVID-19	470	8.8
SARS-CoV-2	416	10.3
Antigen test	74	8.9
Rapid antigen test	68	5.9
RT-PCR	59	13.4
Antigen	41	16.3
Diagnosis	35	14.3
Sensitivity	32	5.8
Coronavirus	31	15.8
Antigen testing	30	6.1
PCR	25	7.3
Point-of-care	23	8.0
Specificity	23	4.8
Public health	21	6.1
Pandemic	20	1.9
Epidemiology	19	3.2
Point-of-care testing	19	16.1
Screening	19	4.6
Self-testing	19	4.3
Rapid antigen tests	18	5.0

## Data Availability

All data is available in the manuscript.
